# Use of Meditation and Cognitive Behavioral Therapies for the Treatment of Stress, Depression and Anxiety in Students. A Systematic Review and Meta-Analysis

**DOI:** 10.3390/ijerph16224394

**Published:** 2019-11-10

**Authors:** Gabriel González-Valero, Félix Zurita-Ortega, José Luis Ubago-Jiménez, Pilar Puertas-Molero

**Affiliations:** Department of Didactics of Musical, Artistic and Corporal Expression, University of Granada, 18071 Granada, Spain; ggvalero@ugr.es (G.G.-V.); felixzo@ugr.es (F.Z.-O.); jlubago@ugr.es (J.L.U.-J.)

**Keywords:** stress, anxiety, depression, students, meta-analysis

## Abstract

The prevalence of mental health problems within students due to high academic demands and learning difficulties is a current challenge the field of education. The aim of this study is to review the scientific literature in order to analyze the effect produced by cognitive-behavioral programs and meditation strategies on stress, anxiety, and depression in students. A further aim is to identify the determinants of treatment success. The bibliographic search was carried out using Web of Science, specifically in the categories of “Education and Educational Research” and “Psychology”, obtaining a sample of 122 articles published between 2007 and 2018. Studies were included which had a pre-experimental or quasi-experimental design and included pre-test and post-test phases. Following application of inclusion criteria, 34 articles were selected for inclusion in a meta-analysis of the random effects of each variable. This obtained an average effect size of −0.41 for stress, −0.37 for anxiety, and −0.30 for depression. Three moderating variables were analyzed, with significant correlations being found for the type of treatment relating to stress (Q = 11.01, df = 2, *p* = 0.004, R^2^ = 0.294) and depression (Q = 6.14, df = 2, *p* = 0.048; R^2^ = 0.436). The stage of education of the individuals was also found to impact upon anxiety intervention success (Q = 13.093 df = 2, *p* = 0.0009, R^2^ = 0.196). Interventions mainly addressed the importance of meditation strategies, mindfulness programs, and cognitive-behavioral therapy to reduce stress, anxiety, and depression in students. This supports the need to increase research at an early age, considering the treatment of mental health as a key factor influencing academic performance and quality of life.

## 1. Introduction

Research in the field of the mind is increasing, with students becoming key objects of study in the field of social sciences and health [1,2]. As students progress through the educational stages they become more prone to suffering from depression and stress [3], largely due to their greater responsibilities, academic demands, and the difficulties they face when developing their learning and other skills. This has an impact on their psychological well-being and can lead to socio-cultural imbalance and deterioration of their mental health [4]. In this sense, Álvarez, Aguilar, and Lorenzo (2012) [5] emphasize factors such as the pace of study, exams, teaching requirements, socio-cultural pressure, competitiveness amongst classmates, and changes to the diet and sleep cycle as severe determinants of mental health problems in students [6].

Stress is considered as a psycho-physiological reaction that occurs in response to felt demands [7]. When dealing with this concept in the educational field, it refers to an overload of tension that is generated by stressful situations or pressure and causes a mental disorder. According to North and Pfefferbaum (2013) [3] negative stress is a generator of attrition, increasing the vulnerability of individuals in childhood and adolescence to suffering anxiety disorders or depressive symptoms. On the other hand, positive stress allows students to face everyday events and is essential for life [8]. Symptoms of stress in childhood and adolescence are conditioned by stressful indicators such as lack of affection, negative family situation, school problems, poor interpersonal skills, and negative physical-body perceptions [9]. In response to stressful factors, if not worked on at an early age, individuals will experience negative emotions in adulthood and on the job, which leads to moral disengagement and psychosocial problems [10].

Another psychosocial factor that alters the mental health of students is anxiety. This is manifested as an exaggerated fear response towards events that are not always identifiable or may be caused by inappropriate situations [11]. This disorder begins during childhood when individuals lack cognitive maturity [2]. The ability to mentally anticipate develops as one progresses through the stages of education. This produces cognitive mistrust that is manifested as unpleasant feelings and physiological changes [12]. Triggers of students’ anxious responses are related to biological and cognitive triggers, the breathing response, the learning process, and academic stress [13]. As a result, anxiety disorders have increased by 14.90% in the general population since 2005 [7,14].

Taking this conceptual approach, depression in students has gone from being an ignored concept, to constituting an entity of educational psychopathology [15], largely because there was an increase of 18.40% cases between 2005 and 2015 [14]. The existence of this factor in early stages is still a matter of debate but it is included amongst the affective educational disorders. Thus, it is understood as a vital psychophysical disorder that includes psychopathological features and corporal alterations [16]. In the educational field, depression leads to a decrease in mood and emotional response to daily activities during which aggressive behaviors are presented and the consumption of harmful substances emerges [17]. Additionally, the depressive condition is characterized by the contemplation of life as a failure and without meaning, and experiences of irritability, tension, and a lack of energy and a sense of reality [18].

Educational psychology has shown interest in different techniques for the treatment of these psychosocial disorders [19,20]. Intervention strategies based on awareness and the promotion of psychological well-being in the educational context have shown a great impact. This is because they focus on experiences of the present moment without judgment, in order to accept without reacting to what appears in the mental field [21,22,23]. Concentrative meditation or Samatha is one technique that has been used. For this, an internal or external object is used as the center of attention, with concentration being moved away from and then returned to the object [24]. Yoga is a strategy that seeks to expand the field of human existence through postures, allowing one to achieve a firm body, a stable mind, and a benevolent spirit [25]. In order to re-establish cognitive processes, tai chi combines slow physical movements with meditation, body and soul awareness, images, and breathing control [26].

According to that presented above, the present meta-analytical research addresses questions about the treatment of stress, anxiety, and depression in students, through meditation or cognitive behavioral programs in order to observe the impact on mental health problems:

- How many interventions have been conducted with students from 2007 to 2018? What kind of meditation programs and/or cognitive-behavioral therapy has been used?
- What effect do different interventions have on the treatment of stress, anxiety, and depression?
- What treatment factors influence students’ stress, anxiety, and depression?

These questions form the basis of the research hypothesis. This assumes that the treatment of stress, anxiety, and depression in students with meditation and/or cognitive-behavioral programs has a positive effect by reducing levels of these mental health problems. Additionally, the number of investigations including this type of treatment is increasing. The length, type of intervention, and educational stage of the individual will be key factors impacting the significance of the effect produced.

Therefore, the purpose of this meta-analysis is to carry out a systematic review of scientific literature in order to analyze the effect of different meditation treatments and/or cognitive-behavioral programs on stress, anxiety, and depression in students at different stages of education (Primary Education, Secondary Education, and University). Thereby, the main objectives are established: (1) analyze the scientific literature of studies including an experimentation based on meditation programs and/or cognitive-behavioral therapies for the treatment of stress, anxiety, and depression in students; (2) calculate the effect of interventions on the variables of stress, anxiety, and depression within the experimental group; (3) identify the determining factors influencing the efficacy of meditation and/or cognitive-behavioral treatments.

## 2. Materials and Methods 

In order to structure the report and ensure integrity of conclusions the PRISMA statement for systematic reviews that incorporate meta-analysis was used [27,28].

### 2.1. Search Strategy

The bibliography search was carried out during December 2018 using Web of Science (WOS) as the main database. In addition, support repositories such as SCOPUS, Psychology and Behavioral Sciences Collection, and PubMed were used. Following initial selection of articles, reference lists were analyzed to identify further articles that related to stress, anxiety, and depression in students, and included implementation programs at different educational stages. The key individual search terms used were “Stress”, “Anxiety”, “Depression”, “Implementation Program”, and “Students”. Boolean operators such as “and” and “or” were also used. The time range was limited to include only articles published in the last decade (2007–2018). References published in English and Spanish were considered obtaining a total of 188 publications. The search was refined to articles published in the categories of “Education Educational Research” and “Psychology”, providing a total of 122 results.

In determining the study population, the research sample was selected based on the following inclusion criteria: (1) scientific articles reporting a program of meditation and/or cognitive-behavioral therapy in students for the treatment of stress, anxiety, and depression within the educational field; (2) studies that use a pre-experimental or quasi-experimental methodological design with pre-test and post-test; (3) investigations reporting statistical results which enable calculation of the effect size of the program implemented; (4) articles published in journals with a peer review process from 2007 to July 2018. To enable studies with a pre-experimental design (those that do not include a control group) to be considered, the effect size was calculated using results of the differences between the means reported in the post-test phase (acts as an experimental group) and the pre-test phase (acts as a control group). In order to comply with the first criterion, a first reading of the title and summary was made. Afterwards, a systematic reading of the full text of articles was carried out in order to examine it was in accordance with the remaining inclusion criteria. Articles that did not present statistical data (qualitative studies) were excluded as they do not allow statistical analysis. In this way, after applying these conceptual, methodological, and statistical criteria, 88 studies were excluded.

### 2.2. Population and Literary Sample

**Figure 1:**
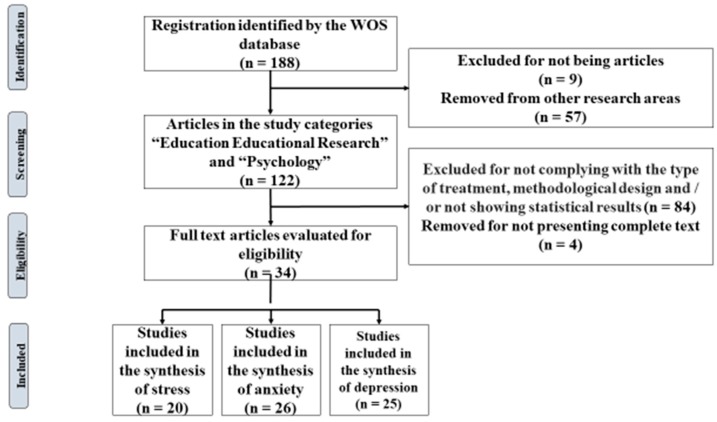
Flow diagram of the selection of articles.

Based on the search strategy, the study population corresponds to 188 scientific articles found in the WOS database. After considering and applying the inclusion criteria, the final meta-analysis sample included 34 scientific articles (Figure 1).

### 2.3. Coding of Articles

The 34 articles that constitute the meta-analysis included 3296 participating students. A total of 22 effect sizes had been calculated for stress treatment, 28 effect sizes for anxiety, and 28 effect sizes for depression. To extract data from the articles, a coding process was followed: (1) author/authors and year of publication; (2) country of intervention (3) educational stage and range or average age of the study subjects; (4) nature of control and experimental group; (5) methodological approach; (6) type of intervention strategy; (7) evaluation instrument; (8) size effect using Cohen’s index d; (9) CI to 95%.

The investigations included in the meta-analysis were coded by each of the authors, in order to check the reliability of coding and the degree of agreement between researchers relating to the selection, extraction of data, and calculation of effect sizes. Degree of agreement in the rating of articles was 90%. This was obtained by dividing the number of coincidences by the total number of categories defined for each study and multiplying it by 100. Selection and codification were carried out independently by all of the study authors, while the calculation of the effect sizes was carried out by two of the authors. In cases of disagreement, agreed solutions were adopted among the coders.

To establish methodological quality of the study, reliability of selection and detection for more than two of the evaluators was determined using the Kappa de Fleiss statistical measure (Kf) [29], whilst the Cohen’s Kappa statistical index (Kc) was used to examine the coding of two authors [30]. A value of Kf = 0.775 was obtained for the selection and extraction of data, which indicates that there is substantial agreement (0.61–0.80). Values of Kc = 0.830 for the calculation of effect sizes, show almost perfect agreement (0.81–1.00) [31].

### 2.4. Effect Size Index

Effect sizes were calculated from the differences of standardized means expressed in units of standard deviation, using the standardized measure Cohen’s d (1988) [32] and the correlation index r, in order to establish post-test differences between groups receiving treatment and control. We then transformed r values into d values. This was done by applying the correction offered by Hedges, Shymansky, and Woodworth (1989) [33] which considers different sample sizes. In addition, variance and the 95% confidence interval (IC) were established for each effect size. Based on the objective of the research, it is important to clarify that negative effect sizes indicate a reduction in students’ mental health problems, whilst positive values indicate that mental health has worsened.

### 2.5. Analysis of Meta-Analysis Data

All statistical analyses were carried out using IBM SPSS^®^ software version 22.0 (IBM Corp, Armonk, NY, USA) and Review Manager 5.3 (Crochrane, London, United Kingdom). Due to the different types of interventions, a random effects meta-analysis model was used which considered intra-study and inter-study variability. Heterogeneity of effect sizes was calculated using the I^2^ index, which evaluates the degree of heterogeneity of individual results. To verify whether or not heterogeneity exists between effect sizes, the Q statistic was calculated. In order to specify examination of a comparison of hypotheses testing the nullity of effects, the Z-bias test was applied. Besides, effect sizes were evaluated through visual inspection of a forest plot. Further, ANOVAs were carried out with the intention of deducing the influence of moderating variables (duration of treatment, educational stage, and intervention strategy).

In order to examine the likely influence of publication bias on the meta-analysis the security number (Ns) was calculated [34]. This compares the study sample, effect size of recovered studies, and the mean effect of studies lost (a value of 0.05 is used). In addition, the number of lost studies is estimated [35] with the assumption of five lost studies for each one published is used, plus a minimum of 10. When the Ns is greater than the number of lost studies, it can be stated that the rigor of the meta-analysis is not threatened by publication bias, however, if the number of lost studies is higher than the Ns, a threat is evident.

### 2.6. Evaluation of the Methodological Quality of the Primary Studies

In order to evaluate the methodological quality of the primary studies, two aspects collected by Botella-Ausina and Sánchez-Meca (2015) [36] must be addressed: (1) quality of the study based on the fact that effect estimates are free of publication bias. A poor evaluation may threaten internal and external validity; (2) quality of the report for which the PRISMA checklist was used [28]. This ensures that the meta-analysis contains relevant data and information enabling its replication.

The 27 items of the PRISMA checklist relate to various aspects of the study [28]. They relate to the title (item 1), abstract (item 2), introduction (items 3 and 4), method (items 5–16), results (items 17–23), discussion (items 24–26), and financial aspects (item 27). The present meta-analysis appropriately addressed the elements that should be included in a report as it meets all of the criteria except item 19 (results on the risk of bias in each study) and item 27 (financing of the meta-analysis).

## 3. Results

### 3.1. Evolution of Scientific Production

**Figure 2:**
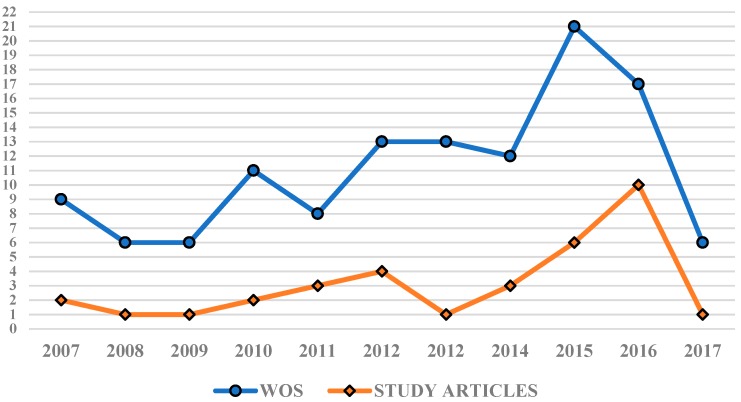
Evolution of scientific production uncovered by WOS and other research databases.

During the period of 2007–2017, 122 articles were published in WOS which referred to the treatment of stress, anxiety, and depression in students. Figure 2 of the evolution of scientific production shows that other research databases provided 27.86% (*n* = 34) of the total scientific production analyzed. There was an upward trend in the publication of articles in WOS over the course of the decade until a maximum was reached in 2015 (*n* = 21), followed by a slight dip in 2016 (*n* = 17). The meta-analysis includes at least one study for each year of this period, with 2015 and 2016 being the years that provide the most articles, with *n* = 6 and *n* = 10 respectively.

### 3.2. Results Generated by Study Interventions Targeting Stress

#### 3.2.1. Study Results According to the Stress Treatment

Table 1 presents results for the treatment of stress in which a total of 1871 students participated in 20 registered studies. A total of 22 effect sizes were calculated with one of the studies carrying out three treatments. The recorded data were statistically significant (*p* < 0.00001) and the weighted mean effect size produced in the experimental groups was $\bar{X}$ = −0.41 (95%CI = −0.52, −0.28). This shows an average effect according to criteria established by Cohen (1988) [32]. The educational stage in which more stress treatments were carried out was university (*n* = 15), followed by Secondary Education (*n* = 5).

**Figure 3:**
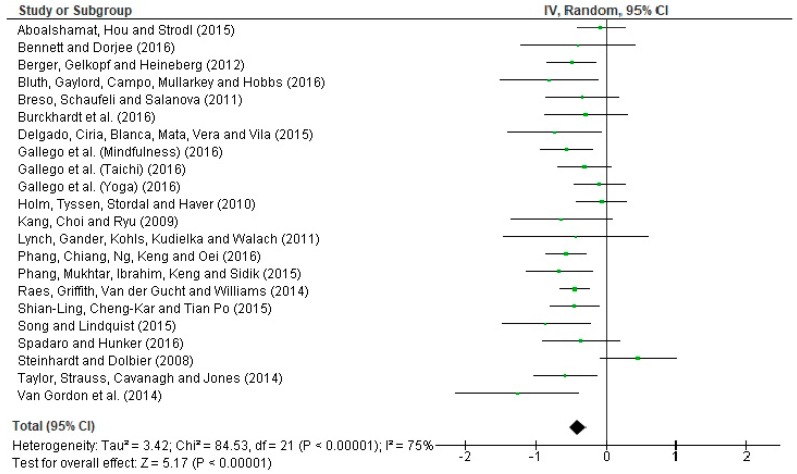
Forest plot for the treatment of stress in students.

A forest plot is shown (Figure 3) in order to interpret the heterogeneity of effect sizes. The Q statistic = 84.53 *(p* < 0.00001) indicates that the results obtained are heterogeneous with respect to the calculated effects size. The I^2^ statistic = 75% suggests high heterogeneity. To examine whether the combined results of the meta-analysis are significant, we used the statistic Z = 5.17 (*p* < 0.00001). This indicates that the combined evidence points to a non-null (significant) effect.

#### 3.2.2. Moderation Analysis

Following identification that real effects are not common amongst all included studies and recognition that the effect sizes obtained are not homogeneous, an analysis of moderating variables was carried out to identify variability. Treatment length, educational stage, and treatment strategy were treated as moderating aspects. Table 2 shows the groupings for each moderator, average effect size, 95% CI, and significance level. 

With regards to treatment duration, short-term interventions show smaller effect sizes ($\bar{X}$ = −0.33, CI = [−0.68, 0.01]) than those produced for interventions lasting more than 9 weeks ($\bar{X}$ = −0.34; CI = [−0.52, −0.15]). Treatments that reported a medium duration present a medium-moderate effect size ($\bar{X}$ = −0.46, CI = [−0.62, −0.30]). With respect to educational stage, results relating to students undertaking Secondary Education obtained higher average effect sizes ($\bar{X}$ = −0.46, CI = [−0.62, −0.29]) than those reported by university students ($\bar{X}$ = −0.38; CI = [−0.53, −0.23]). In consideration of the treatment strategy used, notable differences between categories are noted. Programs based on mindfulness techniques have higher average effect size ($\bar{X}$ = −0.53, CI = [−0.64, −0.42]), compared to programs incorporating body therapies ($\bar{X}$ = −0.19; CI = [−0.45, 0.07]) and cognitive-behavioral programs ($\bar{X}$ = −0.12; CI = [−0.40, 0.16]).

Correlations between the calculated effect sizes and the moderating variables were examined using ANOVAs. The results obtained show that there is a significant effect of treatment strategy with interventions based on mindfulness being associated with greater success (Q = 11.01, df = 2, *p* = 0.004, R^2^ = 0.294). In contrast, the duration of treatment is not associated with TE (Q = 2.66, df = 2, *p* > 0.05), with educational stage similarly not producing associations (Q = 0.42, df = 1, *p* > 0.05). 

#### 3.2.3. Control of Publication Bias.

In the case of stress, a value of Ns = 144 was obtained. This assumes that 144 lost studies with a null effect size are needed, so that when this figure is combined with the studies selected in the present study a combined estimate equal to 0.05 is obtained. The number of lost studies estimated for stress was 110. Thus, we can conclude that the result obtained in the meta-analysis is not threatened by publication bias, since the number of lost studies is less than Ns.

### 3.3. Results Generated by the Interventions of the Study of Anxiety

#### 3.3.1. Study Results Relating to Anxiety Treatment

In the 26 studies that targeted anxiety, 2602 students were included (Table 3) and 28 effect sizes were calculated. The weighted mean effect size of treatment groups was $\bar{X}$ = −0.37 and 95% CI = [−0.50, −0.23], which corresponds to a medium-low effect (Cohen, 1988) [32]. Even so, *p* < 0.00001 shows that the data are statistically significant. The majority of students addressing anxiety took place at university (*n* = 17), with seven studies taking place in Secondary Education and two occurring during Primary Education.

**Figure 4:**
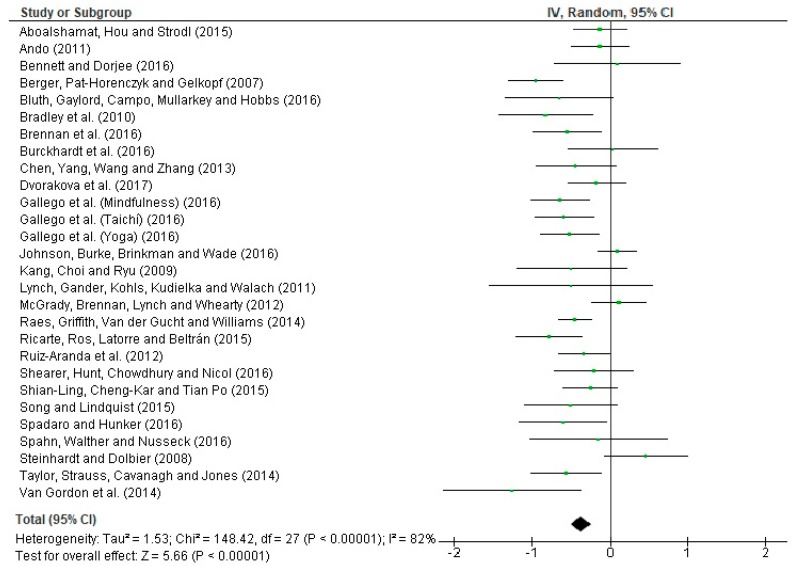
Forest plot of anxiety interventions in students.

The Q statistic = 148.42 (*p* < 0.00001) shows that the results obtained are heterogeneous with respect to the effect sizes calculated in the research. The statistical I^2^ index = 82% indicates that 82% of variance can be attributed to the heterogeneity of results. This confirms high heterogeneity. The Z bias statistic = 5.66 (*p* < 0.00001) shows that the combined results of the meta-analysis are significant. Figure 4 presents a forest plot with the intention of being able to visually interpret the heterogeneity of effect sizes.

#### 3.3.2. Moderation Analysis

In the case of stress, calculated effect sizes are not homogeneous and it is accepted that the real effects are not common in most studies. As a result, an analysis of moderating variables was performed (Table 4). 

With regards to treatment length, short-term interventions show near null effect sizes ($\bar{X}$ = −0.03, CI = [−0.45, 0.39]). Long-term interventions generally obtained small effect sizes with some achieving moderate effects ($\bar{X}$ = −0.36, CI = [−0.56, −0.17]). Finally, interventions lasting between 5 and 8 weeks produced between a medium effect size and a high effect size ($\bar{X}$ = −0.45; CI = [−0.64, −0.26]). In terms of educational stage, studies conducted in Primary Education obtained large effect sizes ($\bar{X}$ = −0.88, CI = [−1.15, −0.61]), while those carried out in Secondary Education produced small average effect sizes ($\bar{X}$ = −0.27, CI = [−0.54, −0.01]). Studies conducted with University students produced medium effects ($\bar{X}$ = −0.40, CI = [−0.55, −0.22]). In consideration of treatment strategy, cognitive-behavioral programs show a small effect size ($\bar{X}$ = −0.27, CI = [−0.61, 0.07]), whilst programs based on mindfulness ($\bar{X}$ = −0.40; CI = [−0.54, −0.24]) and body therapy ($\bar{X}$ = −0.52; CI = [−0.88, −0.15]) show a medium-moderate effect size.

The results show that the educational stage of students influences the likelihood of intervention success, with studies conducted in Primary Education producing a significant correlation (Q = 13.093 df = 2, *p* = 0.0009, R^2^ = 0.196). On the other hand, intervention duration is not related to intervention effect size (Q = 3.20, df = 2, *p* > 0.05), neither was the treatment strategy used (Q = 1.89; df = 2; *p* > 0.05).

#### 3.3.3. Control of Publication Bias

To establish the effect of publication bias on anxiety interventions, the number 140 was determined as the total number of studies lost and Ns was established at 166. As can be seen, the number of lost studies is less than Ns, confirming that the results obtained from this meta-analysis are not threatened by the publication bias.

### 3.4. Results Generated by Intervention Studies for Depression

#### 3.4.1. Study Results of Depression Interventions 

In the 25 studies that reported interventions targeting depression (Table 5), 28 effect sizes were calculated and 2481 students participated. Through assessment of significance findings relating to the data obtained (*p* < 0.00001) we can show that the mean effect size weighted by the intervention groups was $\bar{X}$ = −0.30 and CI = [−0.40, −0.19]. This corresponds to a small but totally relevant effect (Cohen, 1988) [32]. The educational stage that provided the setting for the majority of depression related studies is the university (*n* = 19), whilst the stage of Secondary Education contributed six articles.

**Figure 5:**
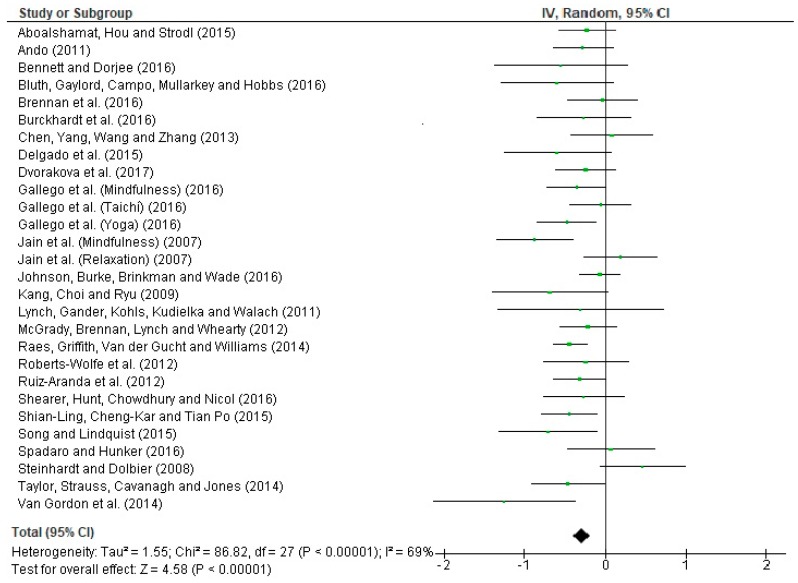
Forest plot of interventions targeting depression in students.

Figure 5 presents a forest plot of anxiety in students. The Q statistic = 86.82 (*p* < 0.00001) which indicates that the data obtained are significant and homogeneous with respect to the effect sizes calculated. The I^2^ index of variance is 69%, which according to Huedo et al. (2006) [61] confirms the emergence of a medium-high heterogeneity. In addition, Z = 4.58 (*p* < 0.00001) which verifies that the meta-analytical results and intervention effects are significant, and the null hypothesis is rejected.

#### 3.4.2. Moderation Analysis

Following moderation analysis of interventions targeting depression (Table 6), a small effect size was produced for short intervention duration ($\bar{X}$ = −0.25, CI = [−0.64, 0.14]), medium duration ($\bar{X}$ = −0.33; CI = [−0.47, −0.19]), and long duration ($\bar{X}$ = −0.25; CI = [−0.38, −0.13]). With regards to educational stage, studies conducted in both Secondary Education ($\bar{X}$ = −0.31, CI = [−0.47, −0.14]) and university ($\bar{X}$ = −0.27; CI = [−0.40, −0.15]) produced a small effect size, though a medium-moderate effect could be achieved depending on the range of effect. With regards to moderation according to the treatment strategy used, cognitive-behavioral programs show a small effect size that was close to a null effect ($\bar{X}$ = −0.06, CI = [−0.27, 0.14]). In body therapy programs the effect size is low ($\bar{X}$ = −0.27, CI = [−0.67, 0.14]), whilst the effect size is medium in mindfulness interventions ($\bar{X}$ = −0.40; CI = [−0.51, −0.27]).

ANOVA results show that treatment strategy moderates the effect achieved in interventions based on mindfulness (Q = 6.14, df = 2, *p* = 0.048, R^2^ = 0.436). However, the duration of the intervention is not associated with the effect sizes (Q = 1.02, df = 2, *p* > 0.05), neither was the educational stage of students (Q = 0.10; df = 1; *p* > 0.05).

#### 3.4.3. Control of Publication Bias

The integrity of results relating to interventions targeting depression is threatened by publication bias as the estimated number of lost studies (135) is slightly higher than the value determined for the Ns (125).

## 4. Discussion

The purpose of the present meta-analysis was to compare the effects produced by different meditation treatments and/or cognitive-behavioral programs on stress, anxiety, and depression in students at different educational stages, in addition to discerning the factors that moderate improvement of these variables. Short-term (less than 4 weeks), medium-term (5–8 weeks), and long-term (more than 9 weeks) interventions were identified. In addition, the different interventions were classified according to cognitive-behavioral therapy programs, mindfulness-based strategies, and body therapy programs. In this way, it was uncovered that interventions targeting a reduction in the stress, anxiety, or depression of students through meditation and/or cognitive-behavioral programs have a positive effect since as they produced improvements. We also emphasize that the number of investigations to include this type of treatment is increasing. Further, the duration and type of intervention and the educational stage of students are key factors that influence the extent of the effect produced.

The 34 articles published in 2007–2017 obtained a medium-moderate effect size for stress (−0.41) and anxiety (−0.37), but a small effect for depression (−0.30) [32]. Comparing these results with other results from similar studies is a complicated task, because no studies have been found that considered all three variables at the same time. Nevertheless, a meta-analysis conducted by Zenner, Herrnleben, and Walach (2014) [64] uncovered evidence of efficacy for interventions based on mindfulness in students. They found that treatment effects were more evident on stress, which is similar to that found in the present study. Attending to the meta-analysis of Guillaumie, Boiral, and Champagne (2017) [65] carried out on university students and nursing professionals, we can also see that similar results to the present study were obtained in the way that a high effect was reported for state anxiety and a moderate effect for depression and trait anxiety.

Regehr, Glancy, and Pitts (2013) [66] analyzed cognitive-behavioral interventions, mindfulness approaches, and relaxation strategies targeting mental health in health students. Again, similar results were obtained to those of the present research. Both this study and the present study found small effect sizes following treatment with cognitive behavioral programs. Mindfulness programs brought about small effects on anxiety and depression, relaxation approaches achieved a large effect on anxiety, a medium effect on depression, and a low effect on stress [1]. Further, a meta-analysis carried out by Werner et al. (2017) [2] can also be considered in which child and adolescent participants were included. This showed low effect sizes for the prevention of depression and anxiety, a finding that agrees with the present results provided for depression.

There is a real interest in introducing stress reduction programs during educational stages. However, there are still few resources that evaluate and improve the effectiveness of mental health treatment within the school setting [67]. Mindfulness Based Stress Reduction (MBSR) approaches are considered to provide acceptable, useful, and beneficial interventions for adolescents. They have been shown to reduce levels of anxiety and depression, which brings with it an improvement in academic performance [19,22,46,48]. These discoveries coincide with Zenner et al. (2014) [64] and Raes et al. (2014) [18] who document improved well-being and academic achievement in students after MBRS treatment. 

Mindfulness is an act in which attention is paid to the present and living the experience of each moment without judgment. In this way, mindfulness-based training reduces symptoms of anxiety and stress in students [21,55]. This evidence coincides with the studies of Jain et al. (2007) [62] and Kang et al. (2009) [45] who examined university students. They described an intervention that emphasized muscle relaxation, diaphragmatic breathing, paying full attention to different parts of the body, non-critical awareness of what arises from moment to moment, and as self-reflection. This led to improvements in problematic behaviors derived from poor mental health. The studies also support the hypothesis that meditation techniques such as yoga, mindfulness, and tai chi contribute to a reduction in emotional stress levels in students [43,47,52,59].

Similarly to this, interventions based on full consciousness have been shown to be effective in reducing depressive symptoms, negative affect, and chronic concern in adolescents [42,57]. Observing the effects produced by treatments, such as the “Strong Minds Condition”, a large average reduction of stress, anxiety, and depression can be seen in adolescents [41,62]. In addition, results of a regression analysis carried out by Bluth et al. (2016) [39] are notable because they have indicated that continuous practice of mindfulness predicts a decrease in depression, anxiety, and perceived stress in adolescents. They also produce improvements in life satisfaction.

Brennan et al. (2016) [54] and McGrady et al. (2012) [56] propose that education and the development of certain skills at university leads to positive behavioral change of students. According to them, interventions should include awareness of breathing, muscle relaxation, coping skills, adequate nutrition, and positive psychology to overcome the problems arising from mental health [49]. Personal development programs in coaching have had a large impact on the psychological control and health of university students. However, it has also been seen that results become less significant as the length of intervention increases [37]. In accordance, Holm et al. (2010) [44] found that levels of stress and mental health improve with the application of personal development programs based on muscle relaxation, whereas students exposed to the treatment program did not improve the parameters of anxiety and depression. Therefore, treatment programs based on self-development and mindfulness considerably reduce levels of stress, anxiety, and depression amongst the student body [20,23].

Cognitive behavioral therapy is considered one of the most popular types of psychotherapy because of its efficacy for anxiety disorders and emotional [68]. In the research literature presented by Breso, Schaufeli, and Salanova (2011) [40], this type of therapy had a positive effect on self-efficacy and commitment, whilst not being related to exhaustion in students. Emotional self-regulation tools that seek to increase emotional self-awareness and improve stress management are noteworthy, since they are effective in reducing negative emotions and anxiety in students [44,53]. Ando (2011) [9] has outlined an initiative for intervention programs. This initiative included the development of social skills with the intention of targeting a specific psychosocial behavior. Results verified that this strategy produces benefits that impact anxiety in students, whilst not producing any positive effects on depression. This fact is supported by some studies that have verified direct associations between depression and anxiety, interpersonal relationships, social self-efficacy, and self-disclosure [15].

Exposure to war is another factor that can be examined in the field of education and alters the mental health of students in countries with a large degree of diversity [69]. Most of the disasters that occur present a great challenge for health, with affected populations seeing a large demand on their mental health services [38]. To confront these circumstances, especially for the reduction of generalized anxiety in childhood, programs that include physical activity, psychoeducational intervention, and coping skills training are recommended [70,71]. As a result of training based on different relaxation and meditation techniques, active participants are able to overcome their concerns and meet the demand for attentional resources associated with problems of stress, anxiety, and depression [50,51,60].

## 5. Conclusions

The present meta-analysis shows that the number of articles reporting interventions targeting these variables in the educational field is increasing. We identified 34 intervention studies published between 2007 and 2018, which reported different durations and addressed the stages of Primary, Secondary, and University Education. We found an average of three articles per year had been published on the topic in the last decade. In relation to the study sample and countries where the interventions were delivered, a total of 14 countries were recorded. The United States was the country with the most interventions, although the European continent accounts for the majority of interventions developed. With regards to the educational stage of the students targeted by interventions to reduce stress, anxiety, and depression, it is observed that the university stage is the most popular setting, followed by Secondary Education. Due to evidence on the emergence of problems at an early age, primary care should be encouraged during early childhood stages to avoid these problems during adolescence and adulthood. 

The effect sizes calculated show that interventions based on cognitive-behavioral programs, self-reflection, and mindfulness-based approaches produce satisfactory and significant results in relation to the reduction of stress, anxiety, and depression in students. This was also corroborated by a good heterogeneity index. Taking into consideration the theoretical foundations of the studies analyzed, meditation (mindfulness, yoga and tai chi), muscle relaxation and breathing, coaching, and cognitive-behavioral therapy are strategies that have shown effectiveness in improving mental health. These aspects should, therefore, be considered as treatment variables in the academic field. It has been shown that programs of medium duration have the best effect on improving mental health in students. Additionally, programs based on mindfulness tend to produce the largest reductions in stress, anxiety, and depression in students. Specifically, there is a greater effect on students in Primary and Secondary Education. 

In short, it is essential to focus attention on how to improve the mental health of students. This implies an increase in the number of interventions in the classroom. The present evidence informs the development of effective activities to improve quality of life and effectiveness and reduce the problems of stress, anxiety, and depression amongst school children. The results obtained in this meta-analytical study have some implications for the treatment of these psycho-social phenomena. We have seen that mindfulness-based strategies are key to reducing mental health problems in students, especially if we start to apply these techniques from early ages. Thus, an opportunity is presented for the participation of teachers in educational research, in which they design intervention programs in order to improve psycho-emotional and educational quality. In addition, there are no studies in the field of educational psychology that jointly address the treatment of these three mental health variables in educational stages spanning youth to young adults. The present meta-analysis, therefore, allows the aforementioned implications to be highlighted.

The main limitation of the present meta-analytical study is the size of the scientific sample, since some educational stages had been scarcely examined. In addition, it was observed that some articles of interventions targeting these variables had been published in journals from other research areas. However, quality of the selected studies is verified by the rigorous review process that is conducted before the paper is accepted for publication. Another aspect that would have broadened the review would have been the inclusion of additional search terms such as “cognitive behavior”, “treatment”, and “mindfulness”. Researchers are encouraged to continue carrying out diagnostic investigations of the student population. A future perspective should be to develop didactic interventions such as those described in the study plans, with the purpose of intervening to change this problematic situation.

## Figures and Tables

**Table 1:** Investigations targeting the stress variable.

Author/s (Year)	Country	Educational Stage (Range or Average Age) *1	Sample (EG-CG) *2	Study Type	Intervention Strategy *3	Instrument *4	Effect Size (d)	95% CI
Aboalshamat, Hou, and Strodl (2015) [37]	Saudi Arabia	UE (20–22)	130 (65-65)	Quasi-experimental (6 weeks)	Self-development programme (HBUSS)	DASS-21	−0.066	[−0.410, 0.277]
Bennett and Dorjee (2016) [19]	Wales	SE (16–18)	24 (11-13)	Quasi-experimental (8 weeks)	MBSR	DASS-21	−0.402	[−1.213, 0.408]
Berger, Gelkopf, and Heineberg (2012) [38]	Israel	SE (12.8 ± 1.0)	154 (107-47)	Quasi-experimental (16 sessions)	EES	DSM IV	−0.480	[−0.830, −0.142]
Bluth, Gaylord, Campo, Mullarkey, and Hobbs (2016) [39]	USA	SE (14–17)	34 (16-18)	Quasi-experimental (6 weeks)	MFY	PSS	−0.823	[−1.524, −0.122]
Breso, Schaufeli, Salanova (2011) [40]	Spain	UE (18–26)	71 (21-50)	Quasi-experimental (4 sessions)	CBT	MBI-SS	−0.330	[−0.842, 0.182]
Burckhardt, Manicavasagar, Batterham, Hadzi-Pavlovic (2016) [41]	Australia	SE (15–18)	46 (24-22)	Quasi-experimental (12 weeks)	Strong Minds Condition (*mindfulness)*	DASS-21	−0.290	[−0.872, 0.291]
Delgado, Ciria, Blanca, Mata, Vera, and Vila (2015) [42]	Spain	UE (21.5 ± 3.94)	41 (27-14)	Quasi-experimental (3 weeks)	MBSR	PSS	−0.745	[−1.410, −0.08]
Gallego, Aguilar, Cangas, Rosado, and Langer (2016) [43]	Spain	UE (18–49)	282 (237-45)	Quasi-experimental (16 sessions)	*Mindfulness* (MBCT)	DASS-21	−0.561	[−0.929, −0.192]
					Yoga		−0.094	[−0.456, 0.266]
					Tai Chi		−0.301	[−0.680, 0.076]
Holm, Tyssen, Stordal, and Haver (2010) [44]	Norway	UE (23.6 ± 3.4)	140 (47-93)	Quasi-experimental (12 sessions/12 weeks)	Self-development and discussion groups	PMSS	−0.061	[−0.412, 0.288]
Kang, Choi, and Ryu (2009) [45]	South Korea	UE (22.47 ± 1.17)	32 (16-16)	Quasi-experimental (8 sessions)	MBSR	PWI-SF	−0.642	[−1.353, 0.068]
Lynch, Gander, Kohls, Kudielka, and Walach (2011) [46]	England	UE (19–46)	16 (10-6)	Quasi-experimental (8 sessions)	MBCUL (MBCT/MBSR)	PSS	−0.448	[−1.472, 0.575]
Phang, Mukhtar, Ibrahim, Keng, and Sidik (2015) [47]	Malaysia	UE (21.04 ± 1.13)	75 (37-38)	Quasi-experimental (5 weeks)	MBSM (MBCT/MBSR)	PSS	−0.669	[−1.135, −0.204]
Phang, Chiang, Ng, Keng, and Oei (2016) [48]	Malaysia	UE (21–25)	104	Pre-experimental (4 weeks)	Mindful-Gym (MBCT/MBSR)	PSS	−0.567	[−0.844, −0.290]
Raes et al. (2014) [18]	Belgium	SE (13–20)	357 (182-175)	Quasi-experimental (8 weeks)	MBCT	DASS-21	−0.443	[−0.653, −0.233]
Shian-Ling, Cheng-Kar, and Tian Po (2015) [49]	Malaysia	UE (21–24)	134 (77-57)	Quasi-experimental (4 weeks)	Mindful-Gym	PSS	−0.174	[−0.517, 0.168]
Song and Lindquist (2015) [20]	South Korea	UE (19.6 ± 1.7)	44 (21-23)	Quasi-experimental (10 weeks)	MBSR	DASS-21	−0.866	[−1.484, −0.247]
Spadaro and Hunker (2016) [50]	USA	UE (18–25)	26	Pre-experimental (8 weeks)	MBSR	PSS	−0.352	[−0.9, 0.195]
Steinhardt and Dolbier (2008) [51]	USA	UE (18–53)	57 (30-27)	Quasi-experimental (4 weeks)	Transforming Lives Resilience Education	CD–RISC	0.467	[−0.06, 0.993]
Taylor et al. (2014) [23]	England	UE (28.61 ± 9.12)	79 (40-39)	Quasi-experimental (8 weeks)	MBCT-SH	DASS-21	−0.576	[−1.026, −0.126]
Van Gordon, Shonin, Sumich, Sundin, and Griffiths (2014) [52]	England	UE (20–42)	25 (14-11)	Quasi-experimental (8 weeks)	MAT	DASS-21	−1.297	[−2.165, −0.429]

Note. * 1 Secondary Education (SE); University Education (UE). * 2 Control group (CG); Experimental group (EG). * 3 “How to Be an Ultra Super Student” (HBUSS); Mindfulness Based Stress Reduction (MBSR); ERASE-Stress extension (EES); Making Friends With Yourself: A Mindful Self-compassion Program for Teens (MFY); Cognitive-behavioral therapy (CBT); *Mindfulness* Based Cognitive Therapy (MBCT); *Mindfulness*-based Coping with University Life (MBCUL); *Mindfulness*-based stress management (MBSM) ** MBCT self-help (MBCT-SH); Meditation Awareness Training (MAT). * 4 Depression, Anxiety, and Stress Scales, 21 item version (DASS-21): Perceived Stress Scale (PSS); Scale of Academic Burnout Syndrome (MBI-SS); Perceived Medical School Stress (PMSS); The psychosocial wellbeing index-short form (PWI-SF); Connor–Davidson Resilience Scale (CD–RISC) ** Perceived Stress Questionnaire (PSQ).

**Table 2:** Analysis of stress moderators.

Study Groups	K *	M *	CI to 95%	*p* *
According treatment duration				
Short duration (0–4 weeks)	5	−0.33	[−0.68, 0.01]	0.26
Medium duration (from 5 to 8 weeks)	10	−0.46	[−0.62, −0.30]	
Long duration (more than 9 weeks)	5	−0.34	[−0.52, −0.15]	
According the educational stage of students receiving treatment				
Secondary Education	5	−0.46	[−0.62, −0.29]	0.52
University	15	−0.38	[−0.53, −0.23]	
According to intervention strategy				
Cognitive-behavioral programs	5	−0.12	[−0.40, 0.16]	0.004 *
Programs based on *mindfulness*	15	−0.53	[−0.64, −0.42]	
Body therapy programs (Yoga-Tai Chi)	2	−0.19	[−0.45, 0.07]	

Note: * Number of interventions (K); average effect size (M); Statistically significant (*p*).

**Table 3:** List of studies addressing anxiety.

Author/s (Year)	Country	Educational Stage (Range or Average Age)*1	Sample (EG-CG)*2	Study Type	Intervention strategy*3	Instrument*4	Effect Size (d)	95% CI
Aboalshamat, Hou, and Strodl (2015) [37]	Saudi Arabia	UE (20–22)	130 (65-65)	Quasi-experimental (6 weeks)	Self-development program (HBUSS)	DASS-21	−0.122	[−0.466, 0.222]
Ando (2011) [9]	Japan	UE (19.1 ± 1.5)	191 (157-34)	Quasi-experimental (11 sessions/11 weeks)	Successful Self Program	Profile of Mood Status	−0.124	[−0.495, 0.246]
Bennett and Dorjee (2016) [19]	Wales	SE (16–18)	24 (11-13)	Quasi-experimental (8 weeks)	MBSR	DASS-21	0.111	[−0.692, 0.914]
Berger, Pat-Horenczyk, and Gelkopf (2007) [38]	Israel	PE (7–11)	142 (70-72)	Quasi-experimental (8 sessions)	OTT manual	SCARED	−0.957	[−1.304, −0.61]
Bluth, Gaylord, Campo, Mullarkey, and Hobbs (2016) [39]	USA	SE (14–17)	34 (16-18)	Quasi-experimental (6 weeks)	MFY	STAI	−0.664	[−1.356, 0.027]
Bradley, McCraty, Atkinson, Tomasino, Daugherty, and Arguelles (2010) [53]	USA	SE (15.3 ± 0.45)	48 (27-21)	Quasi-experimental (12 weeks)	Test Edge	TAI	−0.835	[−1.429, −0.241]
Brennan, McGrady, Lynch, Schaefer, Whearty (2016) [54]	USA	UE (23.3 ± 2.3)	42	Pre-experimental (4 sessions per semester)	Stress management and relaxation strategy	BAI	−0.549	[−0.985, −0.114]
Burckhardt, Manicavasagar, Batterham, Hadzi-Pavlovic (2016) [41]	Australia	SE (15–18)	46 (24-22)	Quasi-experimental (12 weeks)	SMC (*mindfulness)*	DASS-21	0.034	[−0.544, 0.613]
Chen, Yang, Wang, and Zhang (2013) [55]	China	UE (19.5 ± 0.87)	60 (30-30)	Quasi-experimental (7 sessions/1 week)	*Mindfulness* meditation training	SAS	−0.440	[−0.952, 0.071]
Dvorakova et al. (2017) [21]	USA	UE (18.2 ± 0.4)	109 (55-54)	Quasi-experimental (8 sessions/6 weeks)	*Mindfulness* (L2B)	GAD	−0.170	[−0.546, 0.205]
Gallego, Aguilar, Cangas, Rosado, and Langer (2016) [43]	Spain	UE (18–49)	282 (237-45)	Quasi-experimental (16 sessions)	*Mindfulness* (MBCT)	DASS-21	−0.640	[−1.010, −0.269]
					Yoga		−0.519	[−0.886, −0.152]
					Tai Chi		−0.589	[−0.973, −0.204]
Johnson et al. (2016) [22]	Australia	SE (13.63 ± 0.43)	258 (111-147)	Quasi-experimental (12 weeks)	MBCT/MBSR	DASS-21	0.192	[−0.054, 0.439]
Kang, Choi, and Ryu (2009) [45]	South Korea	UE (22.47 ± 1.17)	32 (16-16)	Quasi-experimental (8 sessions)	MBSR	STAI	−0.505	[−1.209, 0.198]
Lynch, Gander, Kohls, Kudielka, and Walach (2011) [46]	England	UE (19–46)	16 (10-6)	Quasi-experimental (8 sessions)	MBCUL (MBCT/MBSR)	HADS	−0.55	[−1.579, 0.479]
McGrady, Brennan, Lynch, and Whearty (2012) [56]	USA	UE (23.4 ± 2.36)	134 (52-82)	Quasi-experimental (8 sessions)	Wellness programs strategies	BAI	0.120	[−0.226, 0.468]
Raes et al. (2014) [18]	Belgium	SE (13–20)	357 (182-175)	Quasi-experimental (8 weeks)	MBCT	DASS-21	−0.443	[−0.653, −0.233]
Ricarte, Ros, Latorre, and Beltrán (2015) [57]	Spain	PE (6–13)	90 (45-45)	Quasi-experimental (8 weeks)	MBI	STATIC	−0.799	[−1.228, −0.369]
Ruiz-Aranda, Salguero, Cabello, Palomera, and Fernández-Berrocal (2012) [58]	Spain	SE (13–16)	147 (78-69)	Quasi-experimental (10 weeks)	Training program (INTEMO Project)	BASC	−0.331	[−0.657, −0.005]
Shearer, Hunt, Chowdhury, and Nicol (2016) [59]	USA	UE (-)	74 (52-22)	Quasi-experimental (4 weeks)	MBSR	STAI	−0.205	[−0.704, 0.294]
Shian-Ling, Cheng-Kar, and Tian Po (2015) [49]	Malaysia	UE (21–24)	134 (77-57)	Quasi-experimental (4 weeks)	Mindful-Gym	DASS-21	−0.250	[−0.594, 0.093]
Song and Lindquist (2015) [20]	South Korea	UE (19.6 ± 1.7)	44 (21-23)	Quasi-experimental (10 weeks)	MBSR	DASS-21	−0.511	[−1.112, 0.089]
Spadaro and Hunker (2016) [50]	USA	UE (18–25)	26	Pre-experimental (8 weeks)	MBSR	HADS	−0.607	[−1.163, −0.051]
Spahn, Walther, and Nusseck (2016) [60]	Germany	UE (22.1 ± 2.3)	21 (13-8)	Quasi-experimental (14 weeks)	Seminars to overcome the MPA	STAI	−0.149	[−1.031, 0.732]
Steinhardt and Dolbier (2008) [51]	USA	UE (18–53)	57 (30-27)	Quasi-experimental (4 weeks)	Transforming Lives Resilience Education	CD–RISC	0.467	[−0.06, 0.993]
Taylor et al. (2014) [23]	England	UE (28.61 ± 9.12)	79 (40-39)	Quasi-experimental (8 weeks)	MBCT-SH	DASS-21	−0.561	[−1.011, −0.111]
Van Gordon, Shonin, Sumich, Sundin, and Griffiths (2014) [52]	England	UE (20–42)	25 (14-11)	Quasi-experimental (8 weeks)	MAT	DASS-21	−1.297	[−2.165, −0.429]

Note. *1 Primary Education (PE); Secondary Education (SE); University Education (UE). *2 Control group (CG); Experimental group (EG). *3 How to Be an Ultra Super Student” (HBUSS); Mindfulness Based Stress Reduction (MBSR); “Overshadowing the Threat of Terrorism” (OTT manual); Making Friends With Yourself: A Mindful Self-compassion Program for Teens” (MFY); Strong Minds Conditions (SMC); *Mindfulness* program Learning to BREATHE (L2B); Mindfulness Based Stress Reduction (MBSR); *Mindfulness* Based Cognitive Therapy (MBCT); *Mindfulness*-based Coping with University Life (MBCUL); *Mindfulness* Emotional Intelligence Training Program (MBI); Music performance anxiety (MPA); MBCT self-help; Meditation Awareness Training (MAT). *4 Depression, Anxiety, and Stress Scales, 21 item version (DASS-21); Screen for Child Anxiety Related Emotional Disorders (SCARED); State-Trait Anxiety Inventory (STAI); The Test Anxiety Inventory (TAI); Beck Anxiety Inventory (BAI); Self-Rating Anxiety Scale (SAS); Generalized Anxiety Disorder Scale (GAD); The Hospital Anxiety and Depression Scale (HADS); State-Trait Anxiety Inventory for Children (STAIC); Behavior Assessment System for Children and Adolescents (BASC); Connor–Davidson Resilience Scale (CD–RISC).

**Table 4:** Analysis of anxiety moderators.

Study Groups	K *	M *	95% CI	p *
According to treatment length				
Short duration (0–4 weeks)	3	−0.03	[−0.45, 0.39]	0.20
Medium duration (from 5 to 8 weeks)	14	−0.45	[−0.64, −0.26]	
Long duration (more than 9 weeks)	9	−0.36	[−0.56, −0.17]	
According to the educational stage of students receiving treatment				
Primary Education	2	−0.88	[−1.15, −0.61]	0.0009 *
Secondary Education	7	−0.27	[−0.54, −0.01]	
University	17	−0.40	[−0.55, −0.22]	
According to intervention strategy				
Cognitive-behavioral programs	8	−0.27	[−0.61, 0.07]	0.39
Programs based on *mindfulness*	18	−0.40	[−0.54, −0.24]	
Body therapy programs (Yoga-Tai Chi)	2	−0.52	[−0.88, −0.15]	

Note: * Number of interventions (K); Average effect size (M); Statistically significant (*p*).

**Table 5:** List of studies targeting depression.

Author/s (Year)	Country	Educational Stage (Range or Average Age)*1	Sample (EG-CG)*2	Study Type	Intervention Strategy*3	Instrument*4	Effect Size (d)	CI to 95%
Aboalshamat, Hou, and Strodl (2015) [37]	Saudi Arabia	UE (20–22)	130 (65-65)	Quasi-experimental (6 weeks)	Self-Development Program (HBUSS)	DASS-21	−0.226	[−0.571, 0.118]
Ando (2011) [9]	Japan	UE (19.1 ± 1.5)	191 (157-34)	Quasi-experimental (11 sessions/11 weeks)	Successful Self Program.	Profile of Mood Status	−0.361	[−0.733, 0.011]
Bennett and Dorjee (2016) [19]	Wales	ES (16–18)	24 (11-13)	Quasi-experimental (8 weeks)	MBSR	DASS-21	−0.566	[−1.385, 0.252]
Bluth, Gaylord, Campo, Mullarkey, and Hobbs (2016) [39]	USA	SE (14–17)	34 (16-18)	Quasi-experimental (6 weeks)	MFY	SMFQ	−0.611	[−1.299, 0.078]
Brennan, McGrady, Lynch, Schaefer, Whearty (2016) [54]	USA	UE (23.3 ± 2.3)	42	Pre-experimental (4 sessions per semester)	Stress management and relaxation strategy	BDI-II	−0.035	[−0.463, 0.392]
Burckhardt, Manicavasagar, Batterham, Hadzi-Pavlovic (2016) [41]	Australia	SE (15–18)	46 (24-22)	Quasi-experimental (12 weeks)	Strong Minds Condition (*mindfulness)*	DASS-21	−0.264	[−0.845, 0.316]
Chen, Yang, Wang, and Zhang (2013) [55]	China	UE (19.5 ± 0.87)	60 (30-30)	Quasi-experimental (7 sessions/1 week)	*Mindfulness* meditation training	SDS	0.086	[−0.419, 0.592]
Delgado, Ciria, Blanca, Mata, Vera, and Vila (2015) [42]	Spain	UE (21.5 ± 3.94)	41 (27-14)	Quasi-experimental (3 weeks)	MBSR	BDI	−0.607	[−1.266, 0.051]
Dvorakova et al. (2017) [21]	USA	UE (18.2 ± 0.4)	109 (55-54)	Quasi-experimental (8 sessions/6 weeks)	*Mindfulness* (L2B)	PHQ	−0.245	[−0.622, 0.131]
Gallego, Aguilar, Cangas, Rosado, and Langer (2016) [43]	Spain	UE (18–49)	282 (237-45)	Quasi-experimental (16 sessions)	*Mindfulness* (MBCT)	DASS-21	−0.353	[−0.717, 0.011]
					Yoga		−0.474	[−0.840, −0.108]
					Tai Chi		−0.056	[−0.432, 0.320]
Jain et al. (2007) [62]	USA	UE (18–61)	81 (51-30)	Quasi-experimental (4 weeks)	*Mindfulness* (MBSR)	DER	−0.842	[−1.384, −0.299]
					Relaxation training		0.202	[−0.335, 0.740]
Johnson et al. (2016) [22]	Australia	SE (13.63 ± 0.43)	258 (111-147)	Quasi-experimental (12 weeks)	MBCT/MBSR	DASS-21	−0.067	[−0.314, 0.178]
Kang, Choi, and Ryu (2009) [45]	South Korea	UE (22.47 ± 1.17)	32 (16-16)	Quasi-experimental (8 sessions)	MBSR	BDI	−0.703	[−1.417, 0.010]
Lynch, Gander, Kohls, Kudielka, and Walach (2011) [46]	England	UE (19–46)	16 (10-6)	Quasi-experimental (8 sessions)	MBCUL (MBCT/MBSR)	HADS	−0.318	[−1.336, 0.700]
McGrady, Brennan, Lynch, and Whearty (2012) [56]	USA	UE (23.4 ± 2.36)	134 (52-82)	Quasi-experimental (8 sessions)	Wellness strategies	BDI-II	−0.214	[−0.562, 0.134]
Raes et al. (2014) [18]	Belgium	SE (13–20)	357 (182-175)	Quasi-experimental (8 sessions)	MBCT	DASS-21	−0.443	[−0.653, −0.233]
Roberts-Wolfe, Sacchet, Hastings, Roth, and Britton (2012) [63]	USA	UE (20.10 ± 2.67)	58 (35-23)	Quasi-experimental (12 weeks)	Samatha and Vipassana	MASQ	−0.247	[−0.775, 0.280]
Ruiz-Aranda, Salguero, Cabello, Palomera, and Fernández-Berrocal (2012) [58]	Spain	SE (13–16)	147 (78-69)	Quasi-experimental (10 weeks)	Training program (INTEMO Project)	BASC	−0.319	[−0.644, 0.007]
Shearer, Hunt, Chowdhury, and Nicol (2016) [59]	USA	UE (-)	74 (52-22)	Quasi-experimental (4 weeks)	MBSR	BDI-II	−0.270	[−0.770, 0.230]
Shian-Ling, Cheng-Kar, and Tian Po (2015) [49]	Malaysia	UE (21–24)	134 (77-57)	Quasi-experimental (4 weeks)	Mindful-Gym	DASS-21	−0.445	[−0.791, −0.098]
Song and Lindquist (2015) [20]	South Korea	UE (19.6 ± 1.7)	44 (21-23)	Quasi-experimental (10 weeks)	MBSR	DASS-21	−0.715	[−1.325, −0.104]
Spadaro and Hunker (2016) [50]	USA	UE (18–25)	26	Pre-experimental (8 weeks)	MBSR	HADS	0.072	[−0.471, 0.616]
Steinhardt and Dolbier (2008) [51]	USA	UE (18–53)	57 (30-27)	Quasi-experimental (4 weeks)	Transforming Lives Resilience Education	CD–RISC	0.467	[−0.06, 0.993]
Taylor et al. (2014) [23]	England	UE (28.61 ± 9.12)	79 (40-39)	Quasi-experimental (8 weeks)	MBCT-SH	DASS-21	−0.465	[−0.912, −0.018]
Van Gordon, Shonin, Sumich, Sundin, and Griffiths (2014) [52]	England	UE (20–42)	25 (14-11)	Quasi-experimental (8 weeks)	MAT	DASS-21	−1.297	[−2.165, −0.429]

Note. *1 Primary Education (PE); Secondary Education (SE); University Education (UE). *2 Control group (CG); Experimental group (EG). *3 “How to Be an Ultra Super Student” (HBUSS); Mindfulness Based Stress Reduction (MBSR); Making Friends With Yourself: A Mindful Self-compassion Program for Teens” (MFY); *Mindfulness* program Learning to BREATHE (L2B); *Mindfulness* Based Cognitive Therapy (MBCT); *Mindfulness*-based Coping with University Life (MBCUL); MBCT self-help; Meditation Awareness Training (MAT). *4 Depression, Anxiety, and Stress Scales, 21 item version (DASS-21); Short Mood and Feelings Questionnaire (SMFQ); Beck depression inventory-II (BDI-II); Self-Rating Depression Scale (SDS); Beck depression inventory (BDI); The Primary Health Questionnaire (PHQ); Daily Emotion Report (DER); The Hospital Anxiety and Depression Scale (HADS); The mood and anxiety symptom questionnaire (MASQ); Behavior Assessment System for Children and Adolescents (BASC); Connor–Davidson Resilience Scale (CD–RISC).

**Table 6:** Moderation analysis of interventions targeting depression.

Study Groups	K *	M *	CI to 95%	p *
According to treatment length				
Short duration (0–4 weeks)	5	−0.25	[−0.64, 0.14]	0.32
Medium duration (from 5 to 8 weeks)	12	−0.33	[−0.47, −0.19]	
Long duration (more than 9 weeks)	8	−0.25	[−0.38, −0.13]	
According to the educational stage of students receiving treatment				
Secondary Education	6	−0.31	[−0.47, −0.14]	0.21
University	19	−0.27	[−0.40, −0.15]	
According to intervention strategy				
Cognitive-behavioral programs	6	−0.06	[−0.27, 0.14]	0.000 *
Programs based on *mindfulness*	20	−0.40	[−0.51, −0.27]	
Body therapy programs (Yoga-Tai Chi)	2	−0.27	[−0.67, 0.14]	

Note: * Number of interventions (K); Average effect size (M); Statistically significant (*p*).
